# Toxicity and Anti-Inflammatory Effects of *Agave sisalana* Extract Derived from Agroindustrial Residue

**DOI:** 10.3390/plants12071523

**Published:** 2023-03-31

**Authors:** Luisa Taynara Silvério da Costa, Julia Amanda Rodrigues Fracasso, Lucas Pires Guarnier, Gustavo Reis de Brito, Daniel Baldini Fumis, Renata Aparecida de Camargo Bittencourt, Aimée Maria Guiotti, Débora de Barros Barbosa, Isabel Cristina Cherici Camargo, Edislane Barreiros de Souza, Pedro de Oliva Neto, Lucinéia dos Santos

**Affiliations:** 1 Department of Biotechnology, School of Sciences and Languages, São Paulo State University (UNESP), Assis 19806-900, SP, Brazil; 2 School of Dentistry, São Paulo State University (UNESP), Araçatuba 05508-000, SP, Brazil; 3 Department of Genetics, Ribeirão Preto Medical School, University of São Paulo, Ribeirão Preto 14049-900, SP, Brazil; 4 Department of Biology, School of Sciences and Languages, São Paulo State University (UNESP), Assis 19806-900, SP, Brazil; 5 Departament of Materials Science and Technology, Bauru School of Science, São Paulo State University (UNESP), Bauru 17033-360, SP, Brazil; 6 Department of Biomedicine, Institute of Health Sciences, University of São Paulo (UNIP), Assis 19813-550, SP, Brazil; 7 Department of Dental Materials and Prosthodontics, Araçatuba Dental School, São Paulo State University (UNESP), Araçatuba 16066-840, SP, Brazil

**Keywords:** inflammation, reproductive system, sapogenins

## Abstract

Background: In several countries, the leaf juice of Agave sisalana (also known as sisal) is widely used topically, especially as an antiseptic, and orally for the treatment of different pathologies. However, in Brazil, which is the largest producer of Agave sisalana, its residue, which represents the majority of its weight, has been thrown away. For this reason, the determination of the pharmacological and toxicological potentials of sisal residue and its possible therapeutic use is seen as a way to contribute to the sustainable development and social promotion of the largest producer of sisal in Brazil, the interior of Bahia State, which is among the poorest areas in the country. Given the scarcity of available scientific studies on the pharmacological and toxicological properties of sisal residue juice, this study aimed to promote the acid hydrolysis of this juice to potentiate the anti-inflammatory effect already described in the literature. Furthermore, it aimed to evaluate the toxicological profile of the hydrolyzed extract (EAH) and to determine its acute toxicity, as well as its side effects on the reproductive aspects of rats. Method: The anti-inflammatory effect of EAH was evaluated in vitro using the induction of hemolysis by hypotonic solution and in vivo in rats using the carrageenan-induced paw edema test and the xylene-induced ear edema test. The acute toxicity, resulting from a single-dose administration, was investigated for some manifestation of toxic symptoms related to motor control and consciousness in rats. At a concentration of 100 mg/kg, by repeated doses, the reproductive toxicity effects of EAH in rats were assessed. Results: In vitro anti-inflammatory activity was positive using the human red blood cell membrane stabilization method. In both in vivo tests used to assess the anti-inflammatory activity, EAH (at three doses) significantly inhibited edema when compared to the control group. At a dose of 50 mg/kg, EAH exhibited a greater effect than indomethacin, a nonsteroidal anti-inflammatory drug with known activity. In vivo toxicological studies have shown that EAH does not present toxic effects when administered orally in a single dose, up to 1000 mg/kg. Finally, EAH promoted a gonadotoxic effect and increased the embryonic mortality rate after implantation. Conclusions: It is suggested that the anti-edematogenic effect of the acid hydrolysis extract from sisal juice is due to the high concentration of steroidal sapogenins. Therefore, this extract can be considered a potential new anti-inflammatory or even an important sapogenin source for the development of steroidal glucocorticoids. However, further studies are needed to elucidate the chemical composition of sisal juice. Regarding toxicology studies, EAH did not show cytotoxic and clastogenic potentials, but it presented a powerful reproductive toxic effect in rats.

## 1. Introduction

Brazil is the world’s largest producer of Agave sisalana (also known as sisal), with 90% of its production localized within the state of Bahia [1]. The state of Bahia has a semi-arid climate and, as a result of this unfavorable climate, the production of sisal has an important social function [2]. The fibers obtained from the plant are commercialized worldwide, which is of great economic importance as a source of highly resistant natural fibers for the manufacture of different industrial products, also employed in the automotive industry and the manufacture of ropes, twine, marine cables, carpets, bags, brooms, upholstery and crafts [3,4,5]. Thus, the existing commercial interest in sisal fibers can be derived from the literature, as well as several studies describing its structure and chemical composition [6,7].

The fiber unraveling of the sisal leaves yields 5% of hard fibers. The remaining residue, which consists of mucilage and juice, is normally discarded in Brazil [8]. In several countries, the juice of Agave sisalana leaves presents great ethnopharmacological importance because it is used as an antiseptic in the topical treatment of skin diseases as well as a poultice on wounds [9,10]. Orally, it is used to treat indigestion, flatulence, jaundice, constipation, and dysentery [11]. 

For this reason, the juice of Agave sisalana stands out for representing an important focus in pharmacological research. Studies have been performed in Brazil to characterize Agave sisalana juice and sanction its therapeutic use; however, these studies are scarce. The isolation and structure determination of five steroidal saponins present in Agave sisalana has been reported to confirm the basis of chemical and phytochemical affirmation that this plant can be used in pharmacological tests [12]. There are numerous pharmacological treatments derived from saponins, such as antifungal, anti-allergic, anti-inflammatory, antibiotic, hepatoprotective, and antitumor activity [8,13,14,15,16]. Moreover, steroidal sapogenins, lipophilic portions of saponins, are the precursors of pharmacologically active steroid anti-inflammatory drugs present in glucocorticoids [17]. When considering the economic importance of these drugs, it is necessary to look for new sources of steroidal sapogenins, as well as to consider the development of more potent phytotherapics with fewer side effects [17,18].

However, due to their detergent action and toxic effect, saponins disrupt erythrocytes and promote cell lysis [17]. Thus, due to the cytotoxicity of sisal and the possibility of this plant being used for the development of phytotherapics, this study evaluated the in vitro cytotoxicity of EAH and the preclinical toxicity by the determination of signs of acute toxicity. In addition, toxicity studies were conducted after repeated dose administration to potential side effects related to the male reproductive system, in addition, several scientific studies with plants that contain steroidal saponins are targets of studies in reproductive toxicology. 

This study aimed to investigate in vitro and in vivo the toxicity and anti-inflammatory activity of the extract, resulting from the acid hydrolysis of Agave sisalana leaves (EAH). Chemical hydrolysis is capable of removing the sugar chain from saponins, allowing the release of its lipophilic portion, the steroidal sapogenins. Although this study is an unprecedented initiative, researching through the technical and scientific works and discarding the usage of sisal juice proved to be challenging. This allowed added economic value, which will surely reflect on the social and health aspects of the population that depends solely on sisal for their livelihood.

## 2. Results

### 2.1. Saponins

Saponins are present in many medicinal and herbal plants around the world [19]. They exhibit a myriad of biological activities, including antifungal, antimicrobial, antiviral, anti-inflammatory, anticancer, antioxidant, and immunomodulatory effects [20]. They can serve as a good starting point for the development of drugs derived from natural products [21].

In our study, according to Table 1, a high amount of Quijalla saponins was reported.

Saponins of the Quillaja class are triterpenoid saponins comprising a hydrophobic chileic acid structure and moieties of hydrophilic sugar. Quillaja saponin products are commercially available, and their composition and properties are recognized, as well as the techno functionality of Quillaja saponins in a variety of food, cosmetic, and pharmaceutical applications [22].

### 2.2. Anti-Inflammatory Activity

The in vitro evaluation showed that all of the treatments used in this assay were significantly different from the negative control (Table 2), which means it is possible to infer that EAH has some anti-inflammatory activity, as it inhibited the occurrence of hemolysis at the concentrations evaluated and resembled the positive control (standard corticosteroid for the treatment of inflammatory pathologies). The HRBC method is often used for the analysis of plant extracts [23], but there are no studies that evaluated extracts from sisal residue for comparisons. However, corroborating the results obtained, the phytochemical screening performed by Araldi et al. [8] proved the existence of steroidal sapogenins (Hecogenin and Tigogenin) through the results of liquid chromatography–mass spectrometry (LC–MS), comparing them with commercial saponin obtained from the bark of the South American soap tree, Quillaja. Thus, the anti-inflammatory activity of EAH is mainly due to its phytochemical composition rich in steroidal sapogenins.

**Figure 1:**
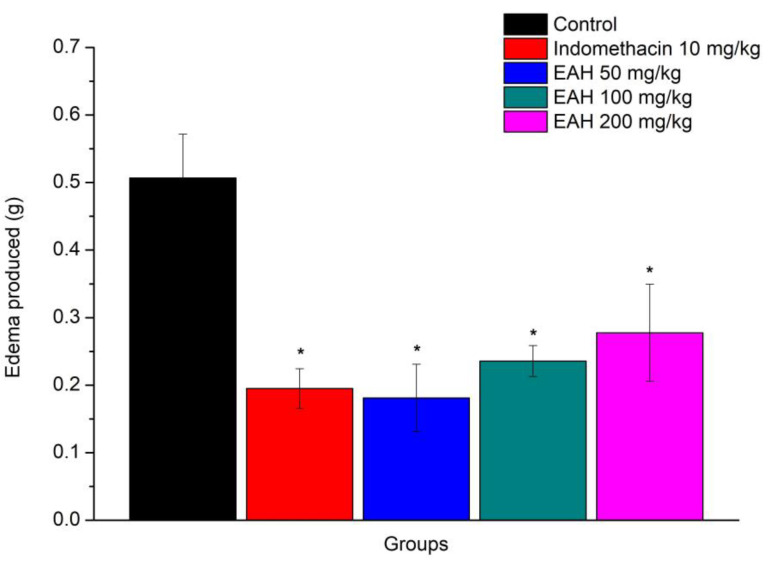
Average (±EPM) of the anti-edematogenic activity of EAH at doses of 50, 100, and 200 mg/kg. The values presented in the graph represent the difference in the volume of water displaced in mL of the right hind paw, * *p* < 0.05 when compared to the control group (*n* = 5 for all groups). ANOVA followed by Duncan’s test.

The results obtained by the prior administration of EAH, in the model of paw edema promoted by carrageenan, are shown in Figure 1 and show the anti-inflammatory action of this extract [F (4, 20) = 6.61, *p* = 0.001]. It was noticeable that EAH demonstrated significant inhibition of edema at all doses employed when compared to the control group (*p* < 0.05). The percentages of inhibition of edema were 64.26%, 53.47%, and 45.20%, respectively, for doses of 50, 100, and 200 mg/kg.

**Figure 2:**
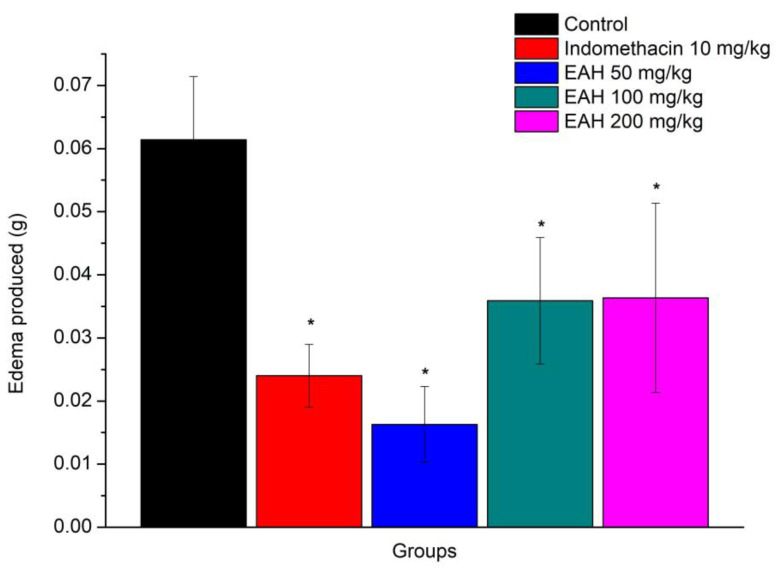
Average (±EPM) of the antiedematogenic activity of EAH at doses of 50, 100, and 200 mg/kg. The values shown in the graph represent the difference in weight between the left and right ear, * *p* < 0.05 when compared to the control group (*n* = 5 for all groups). ANOVA followed by Duncan’s test.

Corroborating previous results, the anti-inflammatory action of EAH was also observed in the xylene-induced ear edema (EAH) model [F (4, 20) = 3.59, *p* = 0.023], Figure 2. Through Duncan’s test, it was noticeable that EAH demonstrated significant inhibition of edema in all doses employed when compared to the control group (*p* < 0.05). The percentage of the inhibition of edema was 73.41%, 41.53%, and 40.80%, respectively, for doses of 50, 100, and 200 mg/kg.

The results showed that the model of paw edema induced by carrageenan and EAH, in the three doses tested presented an anti-edematogenic effect, like indomethacin, used as a positive standard in the experiment. Even at a dose of 50 mg/kg, EAH presented a greater effect than indomethacin, a non-steroidal anti-inflammatory with known activity. The results obtained on the model of ear edema induced by xylene confirmed the anti-edematogenic effect of EAH in all three doses used, and again, at a dose of 50 mg/kg, EAH exhibited a greater effect than indomethacin.

In line with the results of this study, Dunder et al. [13] observed that the oral administration of the hydrolyzed sisal extract at a dose of 500 mg/kg reduced 19.3% paw edema and 51.8% ear edema compared to control groups. In 2013, Dunder et al. [14] reported that the hexane fraction of the hydrolyzed sisal extract, at a dose of 25 mg/kg orally, inhibited 58% of carrageenan-induced paw edema and 62% of xylene-induced ear edema. In the same experiment, dexamethasone, a glucocorticoid used as a positive control, at a dose of 2 mg/kg, promoted a 64% inhibition of paw edema. Mwale et al. [24] demonstrated in 2012 that the aqueous extract of Agave sisalana at doses of 200 and 400 mg/kg caused a significant reduction in carrageenan-induced inflammation in the paws of animals within 2 to 4 h after administration, while 4 h after the administration of carrageenan, the aqueous extract of Agave sisalana at the dose of 400 mg/kg decreased 93.4% of the edema.

Given these studies, the results seem promising, because the dose of 50 mg/kg EAH promoted a paw edema decrease of 64.27%, an anti-inflammatory effect much greater than the results found by Dunder et al. [13] with the hydrolyzed extract and similar to that obtained by the same authors in 2012 with dexamethasone, a potent steroidal anti-inflammatory.

Furthermore, the anti-inflammatory activity of EAH may likely be enhanced a few hours after carrageenan administration, as shown in the study of Mwale et al. [24], or even with its hexane fraction, as suggested by the results of Dunder et al. [14]. That is because sisal is shown to be rich in steroidal sapogenins [25] which represent the lipophilic portion of saponins and have similar anti-inflammatory action to dexamethasone [26].

### 2.3. Acute Toxicity

In the acute toxicity study of the EAH, the administration of doses 250, 500, and 1000 mg/kg proved harmless from the point of incidence of death, preventing the calculation of LD50 for this route. In addition, in the evaluation of the toxic signs of EAH in the first 12 h after administration, and also during the 14 days of observation, the manifestations of these signs were not observed. Thus, through data analysis and using the previously established numeric parameter, a score of 4 was assigned for all aspects of toxicology, indicating full normality in the general state of the animals, their conscience, and their motor control.

Therefore, the results obtained in the acute toxicity study carried show that the extract resulting from the acid hydrolysis of sisal was non-toxic when administered up to 1000 mg/kg orally in rats.

### 2.4. Reproductive Toxicity

Table 3 contains values for the body weight and relative weight of the reproductive organs of rats. In evaluating the results, the C/60 and EAH/60 groups were statistically compared. There was no significant difference (*p* > 0.05) in the body weight and the weight of the testicles, epididymis, and prostate of the animals from the EAH and control groups. The relative weight of the full and empty seminal vesicles was significantly reduced (*p* < 0.05) in the EAH/60 group compared to the C/60 group. Additional analysis would be needed to assess the structure of this glandular tissue and luminal contents to obtain a better understanding of the effect of EAH on the reproductive organ weight. However, in this study, it was found that the weight of the testicles was not affected by treatment, even with the occurrence of histopathological changes.

Testicular tissue analysis revealed that in the C/60 group, the seminiferous tubules presented normal cytoarchitecture with the germinal epithelium composed of several layers of cells and the presence of sperm in the light. In the groups treated with EAH, a loss of the morphological integrity of the seminiferous tubules was detected. These structures exhibited atrophy, a loss of germinative cells in the epithelium, intraepithelial vacuolization, the presence of the formation of multinucleated germ cells, and the presence of immature cells in the light. Furthermore, there was a lack of seminiferous tubules with sperm. Leydig cells were not affected by treatment with the sisal extract.

In the morphometric analysis of the seminiferous tubules (Table 4), the EAH/60 group showed lower values (*p* < 0.05) for the luminal area and tubular diameter when compared to the C/60 group. The area and height of the seminiferous tubular germinal epithelium differed (*p* < 0.05) in the EAH/60 group compared to the C/60 group. These results confirmed the seminiferous tubular atrophy observed in the histological sections of the testicles of the rats treated with the sisal extract.

Treatment with Tribulus terrestris, a plant rich in steroidal saponins, indicated a protective effect against reproductive damage induced by cyclophosphamide in the male reproductive system in mice [27]. In addition, treatment with Ginkgo biloba, another plant that presents steroidal saponin in its composition, showed no toxic effect on rat testicular tissue [28]. In the study with Allium sativum, there was an increase in the number of seminiferous tubules devoid of sperm [29]. In this study, EAH presented several changes in the testicular tissue of animals. The results allow us to conclude that the combination, concentration, and biotransformation of chemical compounds from the sisal juice extract in the body can promote testicular toxicity and impair spermatogenesis.

Table 5 shows the reproductive and fetal parameters. The pregnancy rate was reduced in the EAH/60 group (*p* < 0.05). The analysis of the fetal parameters showed that the paternal treatment with EAH for 60 days increased (*p* < 0.05) the rate of embryo implantation loss, indicating embryonic death. There was no change in the external fetal morphology in the groups treated with EAH, demonstrating no teratogenicity potential of the extract. Although all females became pregnant, the reduction in the pregnancy rate in the EAH/60 group may have occurred due to damage to the fertilizing capacity of the male gamete. The study demonstrated that the chemical constituents of sisal promote negative effects on reproduction and cause early embryonic death. In another study, Viel et al. [15] tested Agave sisalana extract for 30 days consecutive by oral gavage in mice, and the results showed no pathological changes in the ovaries and uterine endometrium but demonstrated the low size and weight of the fetus. Nde et al. [30] suggest that secondary metabolites such as alkaloids and saponins alone or in synergy with other metabolites may interfere in the uterus and, consequently, may be responsible for antifertility effects.

## 3. Materials and Methods

### 3.1. Animals

In the evaluations of the in vivo anti-inflammatory activity, acute toxicity, and reproductive toxicity, 70-day-old male and female Wistar rats (Rattus norvegicus) were used. The animals were maintained in the Animal House of the College of Letters and Science of Assis—UNESP, under controlled temperature (22 °C ± 2 °C) and light (12 h light/dark cycle). Food and water resources were given ad libitum. The experimental protocol followed the Ethical Principles in Animal Research adopted by the Brazilian Society of Laboratory Animal Science and was approved by the Ethics Committee on Animal Use—CEUA (Process 0,142,010—Study of anti-inflammatory activity and acute toxicity; Process 0,252,010—Study of reproductive toxicity).

### 3.2. Human Blood Cells

The evaluation of anti-inflammatory activity was conducted in vitro with 10% red cell homogenate. Ten individuals were used to collect 5 mL peripheral blood samples by venipuncture. After analyzing the participants, according to the research criteria, university students aged 18–25 years of both sexes, who were not taking any medication or toxic substances (alcohol, drugs of abuse, cigarettes) were selected. The Informed Consent Form (TCLE) was prepared following Resolution 196/96 of the National Health Council (CNS) and the experimental protocol was approved by the Ethics in Research (CEP) (Process 12322010).

### 3.3. Plant Material

The crude juice of sisal was obtained from farmers in the municipality of Valente, BA, as defined by the Department of Science, Technology, and Innovation of Bahia (SECTI), by the pressing and filtration of the residue obtained from the shredding process of the leaves. This process was performed at the collection site, and, after, the frozen juice was transported to the Faculty of Science and Letters of Assis, São Paulo State University (UNESP), Brazil. 

The species under study was identified as Agave sisalana in the Herbarium Assisense (HASSI) of the State University of São Paulo (Assis, state of São Paulo) where a voucher specimen was deposited under the number 2597.

### 3.4. Preparation of Extract by Acid Hydrolysis (EAH)

To obtain the EAH used in the assessment of pharmacological and toxicological potentials, the natural juice from sisal was initially subjected to sulfuric acid until reaching pH 0.4–0.8 and heated to a high temperature (120 °C) for 150 min. Thereafter, the resulting solution was centrifuged, and the precipitate obtained was dried at 50 °C. The preparation of EAH was performed by diluting the dried precipitate in distilled water.

### 3.5. Phytochemical Screening

The saponin content was measured according to Hiai et al. [31]. Into a test tube was pipetted 0.25 mL of EAH (100, 200, 400, and 600 ug/mL), 0.25 mL of vanillin 10% (*w/v*) fresh, and 2.5 mL H_2_SO_4_ 72% (in cold temperature). The mixture was heated in a water bath for 10 min at 60 °C and cooled. The absorbance of the mixture was measured at a wavelength of 544 nm. Saponin Quillaja (CAS 8047152, Sigma Aldrich^^®^^, St. Louis, MO, USA) was used as the reference standard. The results of the isolated extracts were expressed considering the concentration of saponins per 100 g of dry extract.

### 3.6. Evaluation of the Anti-Inflammatory Activity

#### 3.6.1. In Vitro

The method proposed by Ananthi and Chitra [23], with some modifications, was used for the evaluation of the human red blood cell membrane stabilization (HRBC). The test reaction was performed with the addition of 2 mL of hyposaline solution (0.18%), 1 mL of sodium phosphate buffer (0.1 M, pH 7.4), 1 mL of the analyzed samples (EAH 100μg/mL, 200 μg/mL, 500 μg/mL, 1000 μg/mL and 2000 μg/mL, all dissolved in hyposaline solution) and 0.5 mL of HRBC solution. In the control groups, the following substitutions were performed: in the negative control, the analyzed samples were replaced by a pure hyposaline solution (0.18%) for the production of hemolysis. On the other hand, in the positive control, the analyzed samples were replaced by dexamethasone (100 μg/mL), dissolved in a hyposaline solution (0.18%) to prevent hemolysis. 

The density of hemoglobin contained in the solution was measured in a UV–Vis spectrophotometer at 560 nm. The percentage of membrane stabilization activity (protection) was determined according to the below formula:

Membrane stabilization activity (%) = [(E_0_ − E_T_)/E_0_] * 100 (1)

where E_0_ represents the mean absorbance value of the negative control group; E_T_ represents the mean absorbance value in the treated groups.

#### 3.6.2. In Vivo

The anti-inflammatory effect of EAH was assessed by the methods of carrageenan-induced paw edema and xylene-induced ear edema. In both methods, the different treatments were performed using a single oral dose. At the beginning of the treatment, the rats were randomly divided into five experimental groups (*n* = 5/group): a negative control, in which the animals were treated with distilled water at 5 mL/kg of body weight; a positive control, in which the animals were treated with indomethacin (Merck^®^, Darmstadt, Germany) at a dose of 10 mg/kg of body weight; and EAH/50, EAH/100, and EAH/200, in which the animals were treated with sisal extract at a dose of 50 mg/kg, 100 mg/kg, and 200 mg/kg, respectively.

The carrageenan-induced paw edema (EPC) method was developed according to the model proposed by Winter et al. [32]. EPC in the different treatments was performed 30 min before subplantar injection in the right hind paw of the animal with 0.1 mL of 1% carrageenan (Sigma Aldrich^^®^^, St. Louis, MO, USA). Leg volume readings, with the aid of a plethysmometer (UGO—BASILE), were performed before carrageenan injection and 60 min after administration. The value of edema resulted from the difference between the paw volume after carrageenan injection with 1% (Vf), and the paw volume before the carrageenin injection (Vi), or Vf - Vi.

The xylene-induced ear edema (EOX) method was conducted according to the model proposed by Lee et al. [33]. In all of the treatments, EOX was conducted 30 min before the injection of 0.03 mL of xylene (Merck^®^, Darmstadt, Germany) on the anterior and posterior surfaces of the right ear. The left ear was considered as a control. Two hours after the application of xylene, the rats were sacrificed and both ears were removed. Circular ear discs 7 mm in diameter were formed and weighed. The value of edema was calculated using the difference in weight between the ear injected with xylene and the control.

In the two methods performed to assess the anti-inflammatory activity of EAH, the percentage of inhibition was calculated using the following formula:

Inhibition (%) = [(E_0_ − E_T_)/E_0_] * 100 (2)

where E_0_ is the average value of ear edema in the control group and E_T_ represents the average value of ear edema observed in the treated group.

After evaluation, the rats of each experimental group were induced to death through the use of an excessive dose of the anesthetic thiopental (Thiopentax^®^, Cristália, São Paulo, Brazil).

### 3.7. Evaluation of Toxicity

#### 3.7.1. In Vivo Acute Toxicity

To evaluate the acute toxicity, the different treatments were performed in single oral doses. This study aimed to determine the median lethal dose (LD50) of EAH capable of promoting the death of 50% of animals by OECD guideline 425. Therefore, decreasing doses of EAH in a 1:2 ratio were used, based on doses higher than those determined in the study of anti-inflammatory activity, as therapeutic and compatible with the volume determined for the administration of the extract by gavage in up to 2 mL. A lower dose would not cause death in the livestock population in a period of 48 h after the end of treatment and should be closer to the therapeutic dose. At the beginning of the treatment, the animals were randomly divided into five experimental groups (*n* = 6/group, 3 males and 3 females): negative control, in which the animals were treated with distilled water at 5 mL/kg of body weight; EAH/250, EAH/500 and EAH/1000, which animals were treated with sisal extract at a dose of 250 mg/kg, 500 mg/kg and 1000 mg/kg, respectively.

In addition, to better evaluate the acute toxicity of EAH, after dosing for the first 12 to 15 h, the evaluations were taken every 30 min, 60 min, and 4 h and were evaluated for expressions of some toxic signs. From the 24th hour of administration, the evaluation occurred only once a day for up to 14 days. The animals that died were analyzed according to behavioral and physiological aspects, which are divided into two categories: 1—Motor and State of Consciousness, where the locomotion, tremor, mood, and state of consciousness of the animals were evaluated; 2—General State, assessing the occurrence of salivation, dyspnea, diarrhea, erythema, edema, changes in hair and skin, eye irritation, and mucosal bleeding. The numeric parameters used to evaluate the expression of these aspects were: 7: strongly increased; 6: moderately increased; 5: slightly increased; 4: normal; 3: slightly reduced; 2: moderately reduced; 1: massively reduced; 0: absent.

Special toxicity studies must be performed when there is the possibility of developing the continuous use of herbal medicines. Thus, the genotoxic of EAH and its side effects on reproductive aspects were evaluated. Considering that the concentrations of EAH showed therapeutic effects, as well as the complexity of the tests used, only the intermediate dose of 100 mg/kg of body weight was used in these studies.

#### 3.7.2. Evaluation of Reproductive Toxicity

The rats were randomly divided into two experimental groups (*n* = 12/group), as follows: Control/60, in which animals received distilled water (orally) for 60 consecutive days, and EAH/60, in which animals were treated daily with sisal extract 100 mg/kg for 60 consecutive days. 

After the treatment period, six rats in each group were induced to die by an overdose of thiopental anesthesia (Thiopentax^®^, Cristália, São Paulo, Brazil) via the intraperitoneal route. Their reproductive organs were collected and weighed. The remaining animals (*n* = 6 per group) were mated with untreated females to assess their reproductive performance and fetal parameters.

The tests of each animal were prepared by the usual histological examination. The material was fixed in alcoholic Bouin’s solution, dehydrated in increasing ethanol solutions, diaphanized in xylene, and embedded in Paraplast^^®^^ (St. Louis, MO, USA). Sections of 5 μm thickness, stained by hematoxylin-eosin (HE) and Mallory trichrome, were analyzed by light microscopy for histopathological and morphometric tissue evaluation.

For the morphometric analysis of the test, 10 cross-sections of seminiferous tubules/animal/group were randomly selected to measure the tubular area, lumen area, the height of the seminiferous epithelium, and tubular diameter. The analysis was performed in an optical microscope model Zeiss Axio Scope A1—(Zeiss^®^, Dublin, CA, USA) coupled with the Axio Vision image system scanner. The tubular luminal areas were expressed in μm^2^, and the height and diameter of the tubular epithelium were expressed in μm. 

For the analysis of the reproductive performance and fetal parameters, each rat (*n* = 6 per group) was mated with a female that did not undergo any treatment, in a ratio of 1:1. Confirmation of pregnancy was performed by the presence of sperm in the vaginal smear, which was considered the first day of gestation (DG1). At DG19, laparotomy was performed to obtain reproductive and fetal parameters. The females were anesthetized with 40 mg/kg of Cetamin^®^ (Syntec, São Paulo, Brazil) and 20 mg/kg of Calmiun^®^ (Agener, São Paulo, Brazil), by the intramuscular route. After the exposure of the uterine horns and ovaries, the following records were obtained: the number of implants, the number of gravitic lutea, the number of fetuses in the litter, the fetal viability, and the number of resorptions. The litter weight and placental weight were also obtained. Pre-implantation and post-implantation loss rates, fertility, and pregnancy rates were calculated [8]. The fetuses were analyzed according to their external morphology with the use of a stereomicroscope.

### 3.8. Statistical Analysis

For the statistical analysis of the study, data from each of the assessments was employed using BioEstat 5.0 (Mamirauá Institute, Tefe, Brazil), and the results were discussed at the 5% level of significance.

In the evaluation of anti-inflammatory activity, data were subjected to one-way ANOVA, with treatment as an independent factor. Where appropriate, multiple comparisons were performed by Tukey’s and Duncan’s tests.

In the study of reproductive toxicity, depending on the variable, parametric analysis ANOVA or Kruskal–Wallis was applied, complemented by the Tukey test and Dunn, respectively. The fertility and pregnancy rates in each experimental group were analyzed using the chi-square test (X2)

## 4. Conclusions

Although this extract showed excellent anti-inflammatory activity with an important source of sapogenin for the development of steroidal glucocorticoids, further studies are needed to elucidate the chemical composition of sisal juice, as well as to improve existing studies to maximize the results obtained.

Regarding toxicological studies, EAH did not show cytotoxic and clastogenic potential, but it showed a powerful reproductive toxic effect on rats, especially when administered for a longer period. Thus, to complement the results obtained, new experiments should be performed to determine the median lethal dose of EAH and elucidate its toxicological action on reproductive parameters, especially in other experimental models.

## Figures and Tables

**Table 1:** Average ± SD of saponins equivalent to the standard g/100 g of dry extract.

Saponins	49.24 ± 0.07 EPS g/100 g

**Table 2:** Average ± SD of the percentage of protection against hemolysis for each treatment group. Where NC—Negative control, PC—Positive control, E1—EAH 100 μg/mL, E2—EAH 200 μg/mL, E3—EAH 500 μg/mL, E4—EAH 1000 μg/mL, and E5—EAH 2000 μg/mL.

Treatment	Protection Against Hemolysis (%)
NC	0.00 ± 0.00
PC	99.03 ± 0.03 *
E1	94.07 ± 0.07 *
E2	95.9 ± 0.10 *
E3	97.53 ± 0.16 *
E4	97.88 ± 0.14 *
E5	98.47 ± 0.06 *

Asterisk (*) indicates a significant difference (*p* < 0.05) concerning the negative control (NC). One-way ANOVA was followed by Tukey’s post hoc test.

**Table 3:** Body and organ weights in the experimental groups.

Parameters	Experimental Groups (*n* = 6/Group)	
	C/60	EAH/60
Body weight (g)	464.2 ± 54.3	454.2 ± 51.7
Testes weight (g%)	0.70 ± 0.11	0.69 ± 0.05
Epididymis weight (g%)	0.29 ± 0.04	0.30 ± 0.07
Prostate weight ^#^ (g%)	0.22 ± 0.09	0.13 ± 0.05
Full seminal vesicles weight (g%)	0.46 ± 0.07	0.31 ± 0.06 *
Empty seminal vesicles weight (g%)	0.28 ± 0.08	0.16 ± 0.03 *

* Statistical difference between the groups EAH/60 and C/60 (*p* < 0.05). Values are expressed as mean ± standard deviation (ANOVA, Tukey test); ^#^ Values are expressed as median ± interquartile deviation (Kruskal–Wallis, Dunn test).

**Table 4:** Effect of treatment with EAH on the testicular morphometric parameters.

Parameters	Experimental Groups (*n* = 6/Group)	
	C/60	EAH/60
Tubular area (µm^2^)	66,218.0 ± 11,785.0	62,446.0 ± 12,148.0
Luminal area (µm^2^)	16,762.0 ± 10,422.0	16,622.0 ± 11,420.0 *
Tubular diameter (µm) ^#^	286.7 ± 41.5	278.3 ± 40.6 *
Seminiferous epithelium height (µm)	70.8 ± 13.9	58.7 ± 12.8 *

Values are expressed as mean ± standard deviation (ANOVA, Tukey test); ^#^ Values are expressed as median ± interquartile deviation (Kruskal–Wallis, Dunn test). * *p* < 0.05 in the comparison C/60 vs. EAH/60.

**Table 5:** Effect of Agave sisalana on the reproductive and fetal parameters.

Parameters	Experimental Groups (*n* = 6/Group)	
	C/60	EAH/60
Fertility Index (%)	100	100
Gestation Index (%)	100	66.7
Body weight of dams (g)	362.6 ± 0.4	402.6 ± 0.4
Gravid uterus weight (g)	46.3 ± 0.2	47.5 ± 0.6
Litter size	12.6 ± 0.2	12.2 ± 0.3
Litter weight (g)	22.8 ± 3.3	24.7 ± 6.9
Placental weight (g)	5.8 ± 1.0	6.7 ± 0.9
Fetal viability (%)	100	100
Pre-implantation loss (%)	0	0
Post-implantation loss (%) ^#^	1.25 ± 2.8	12.4 ± 2.9 *

Values are expressed as mean ± standard deviation (ANOVA, Tukey test); ^#^ Values are expressed as median ± interquartile deviation (Kruskal–Wallis, Dunn test). * *p* < 0.05 in the comparison C/60 vs. EAH/60.

## Data Availability

The data that support the findings of this research are available from the corresponding author upon reasonable request.
